# Challenges and Optimization of Percutaneous Coronary Interventions for Coronary Bifurcation Lesions

**DOI:** 10.31083/RCM42749

**Published:** 2026-01-16

**Authors:** Xia An, Zhilu Qin, Zengduoji Ren, Weipeng Zhao, Chunying Fu, Lina Dong, He Lv, Xinyu Li, Qiang Fu

**Affiliations:** ^1^Department of Cardiology, The People’s Hospital of Liaoning Province (The People’s Hospital of China Medical University), 110016 Shenyang, Liaoning, China

**Keywords:** PCI, CBL, challenges, strategies

## Abstract

The complex anatomy of coronary bifurcation lesions (CBLs) remains a major challenge in percutaneous coronary interventions (PCIs). Currently, the single-stent strategy offers procedural simplicity; however, this strategy carries a higher risk of side-branch occlusion. Conversely, the two-stent technique improves branch coverage but is associated with increased risks of metal carina formation and late stent thrombosis. This article reviews the technical key points and indications of the provisional stent, T-stent, Crush, and Culotte techniques. Moreover, this article focuses on discussing the core challenges of different methods according to anatomical characteristics, post-dilatation stent morphology, and procedural variability of lesions during PCI. Furthermore, corresponding optimization strategies were explored to guide individualized treatment of CBLs using the Visual Risk Prediction of Side-branch Occlusion in Coronary Bifurcation Intervention (V-RESOLVE) score, functional assessments, and intracoronary imaging combined with the DEFINITION criteria.

## 1. Introduction

Coronary bifurcation lesions (CBLs) account for about 1/5 of all cases of percutaneous coronary intervention (PCI) [1, 2, 3]. Compared with non-bifurcation PCI, bifurcation PCI is associated with higher procedural complication rates, increased restenosis, and poorer clinical outcomes [4]. The unique hemodynamic characteristics of vascular bifurcation and the resultant features in endothelial shear stress make these regions prone to atherosclerosis. Moreover, the anatomic variability of bifurcation lesions (e.g., plaque burden/distribution, bifurcation angle, vessel diameter, and lesion location) brings many challenges to interventional treatment [5], such as plaque shift-induced side branch occlusion and in-stent restenosis. Although advances in drug-eluting stents and intracoronary imaging have improved outcomes, optimal stenting strategies remain controversial: While single-stent techniques (simpler and more feasible) are often preferred over two-stent approaches, they carry a higher need for bailout stenting in complex cases. Conversely, two-stent techniques (e.g., for severe ostial disease, diffuse lesions, or high risk of compromise) improve branch coverage but introduce risks of metal carina formation and late stent thrombosis [6]. The central challenge lies in moving beyond empirical decision-making to establish a multi-dimensional strategy integrating anatomic features, functional assessment, and intracoronary imaging guidance [7].

This article reviews the technical key points and indications of provisional stenting, T-stenting, Crush, and Culotte techniques, highlighting their core challenges and optimization strategies. Additionally, we discuss emerging technologies such as: Drug-coated balloons (DCB), Bioresorbable scaffolds, BioMime™ branch sirolimus-eluting coronary side-branch stent [8], and the R-One robotic system for percutaneous coronary intervention [9].

## 2. Definition and Evaluation

### 2.1 Definition and Medina Classification

**Fig. 1.**
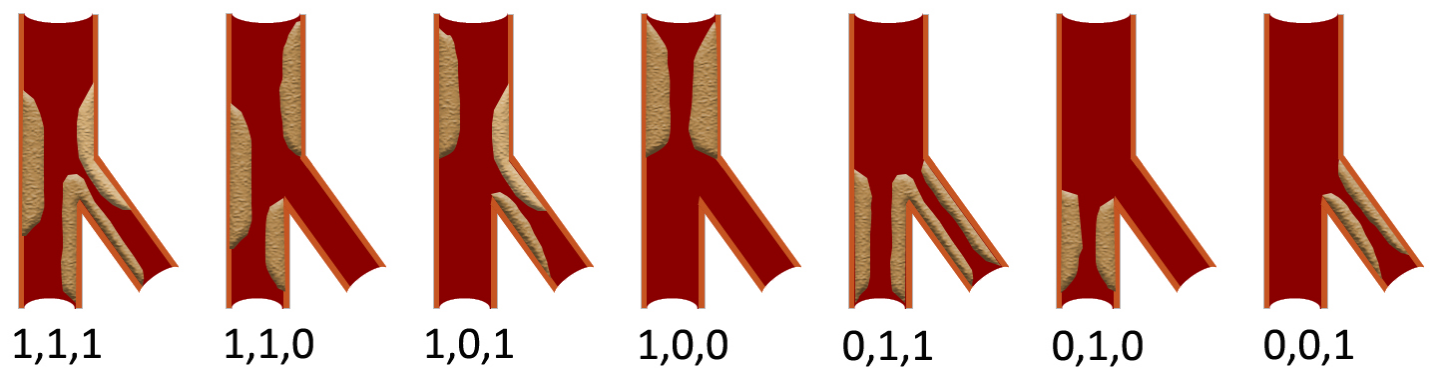
**Schematic diagram of the Medina classification**. The three numbers respectively indicate whether there is significant stenosis (≥50% lumen stenosis) in the proximal main bifurcation, distal main bifurcation and side branch involved by the bifurcation lesion. Among them, 1: There is significant stenosis in this segment; 0: There is no significant stenosis in this segment.

CBLs are defined as stenosis ≥50% located in any segment of the proximal main vessel (pMV), distal main vessel (dMV), or ostium of the side branch (SB). The Medina classification, recognized by major institutions such as the European Bifurcation Club, is the most widely used classification [10, 11] (Fig. 1). It divides CBLs into three segments: pMV, dMV, and SB. Each segment is assigned a binary value (1 or 0) based on whether there is >50% stenosis. When both the main vessel (MV) and SB have >50% stenosis, it is defined as “true CBLs” (Medina classification: 1,1,1; 0,1,1; 1,0,1), while the rest are classified as “false CBLs” (Medina classification: 1,1,0; 1,0,0; 0,1,0; 0,0,1) [12]. True CBLs, due to their anatomic complexity (e.g., asymmetric plaque distribution, variable bifurcation angles), significantly impact the prognosis of PCI and are often associated with poorer clinical outcomes. Compared with non-true CBLs, PCI for true CBLs carries a significantly higher risk of major adverse cardiovascular events (MACE), particularly in left main bifurcation cases [3, 13]. Consequently, these lesions need greater clinical attention and tailored management strategies.

### 2.2 Definition of Complex CBLs

The complexity of CBLs varies due to factors such as the diameter of the stenosis, the length of the lesion, the bifurcation angle, the diameter of the vessels, and the specific pathology of MV and SB lesions. Individualized treatment strategies and optimization of techniques are key to ensuring the success of interventional treatment for CBLs. Currently, complex CBLs are defined according to the DEFINITION criteria [14, 15] (Table 1).

**Table 1.** **The DEFINITION criteria**.

Major criteria (For left main bifurcation): SB diameter stenosis ≥70% and SB lesion length ≥10 mm;
Major criteria (For non-left main bifurcation): SB diameter stenosis ≥90% and SB lesion length ≥10 mm;
Minor criteria: Greater than mild calcification;
Major criteria: Multiple lesions;
Major criteria: Bifurcation angle <45° or >70°;
Major criteria: MV reference diameter <2.5 mm;
Major criteria: Thrombus-containing lesions;
Major criteria: MV lesion length ≥25 mm;
A complex lesion was defined as meeting one of the major criteria plus 2 of the minor criteria.

Notes: MV, main vessel; SB, side branch.

### 2.3 Occlusion Risk Stratification of SB

In the selection of interventional treatment strategies for CBLs, predicting the risk of SB occlusion is one of the key challenges faced by operators. The Visual Risk Prediction of Side-branch Occlusion in Coronary Bifurcation Intervention (V-RESOLVE) scoring system can assess the risk of bifurcation occlusion [16, 17]. This scoring system takes into account various risk factors of different degrees, with a V-RESOLVE score of ≥12 defined as high risk for SB occlusion, as shown in Table 2.

**Table 2.** **The V-RESOLVE scoring system**.

Risk factors	Level	Point
MV TIMI flow grade before stenting	TIMI 3	0
	TIMI 2	6
	TIMI 1	11
	TIMI 0	17
Plaque distribution	at the opposite side of SB	0
	at the same side of SB	1
Pre-procedural diameter stenosis of bifurcation core (%)	<50	0
	≥50; <70	2
	≥70	3
Diameter stenosis of the SB before MV stenting (%)	<50	0
	≥50; <70	4
	≥70; <90	6
	≥90	7
Bifurcation angle (°)	<70	0
	≥70; <90	4
	≥90	6
Diameter ratio between MV/SB	<1.0	0
	≥1.0; <1.5	2
	≥1.5; <2.0	6
	≥2.0	9

Note: SB, side branch; TIMI, thrombolysis in myocardial infarction; MV, main vessel; V-RESOLVE, Visual Risk Prediction of Side-branch Occlusion in Coronary Bifurcation Intervention.

## 3. Key Technologies

### 3.1 Jailed Wire Technique (JWT)

By retaining the SB guidewire during the release of the MV stent [18], it aims to provide a pathway for subsequent rescue operations on the SB [19]. If the SB flow becomes compromised, this wire may serve as a guidewire to facilitate balloon or stent recrossing into the SB ostium. However, it cannot effectively prevent compression or obstruction of the SB ostium, which may occur due to plaque shift or carina shift toward the SB during MV stent expansion [20].

### 3.2 Jailed Balloon Technique (JBT)

An undeployed balloon is pre-positioned in the SB. Following MV stent deployment, the SB balloon is inflated at low pressure (4–6 atm) to maintain ostial patency and subsequently withdrawn. This technique reduces plaque shift through its physical barrier effect, provides a landmark for SB wire re-entry, and significantly mitigates the risk of acute SB occlusion, establishing it as the superior branch protection strategy in CBLs interventions [21, 22].

### 3.3 Rewire Technique 

Rewire technique refers to the critical procedural step of re-crossing a guidewire through the stent cell into the true lumen of the SB after the deployment of the first stent (either in the MV or SB). In CBLs interventions, this technique is pivotal for dual-stent strategies (e.g., Culotte, Crush, double-kissing crush (DK-crush)), directly influencing SB patency and long-term clinical outcomes [23].

### 3.4 Proximal Optimization Technique (POT)

POT is a critical approach in the treatment of CBLs [3, 24]. During the procedure, a non-compliant balloon shorter than the proximal stent coverage is employed, with a diameter matching the proximal reference vessel in a 1:1 ratio [24]. The balloon is then inflated to ensure optimal stent apposition. POT significantly facilitates the passage of branch guidewires and balloons by correcting inadequate stent apposition in the pMV segment, preventing subsequent guidewires from passing beneath the stent and promoting stent strut separation at the branch ostium. Key considerations for POT implementation include: (1) Balloon length should cover the entire pMV stent segment. If the balloon is too short, the procedure must be performed in multiple steps. (2) The length must not exceed the pMV stent segment to avoid proximal stent edge dissection. (3) The distal shoulder of the balloon should be positioned just proximal to the carina [23]. Overly proximal placement may result in insufficient stent expansion in the carina region, while overly distal placement risks carina shift.

### 3.5 Kissing Balloon Inflation (KBI)

​KBI is a pivotal technique in the interventional management of CBLs, particularly when both MV and SB require intervention [5, 25]. By simultaneously inflating balloons in the MV and SB to create a “kissing” configuration at the bifurcation core, KBI offers several mechanistic advantages [21, 26]: (1) optimizing stent apposition to minimize strut coverage over the SB ostium; (2) correcting MV stent deformation caused by SB compression during branch dilation; (3) reducing the risk of SB occlusion caused by plaque shift/carina displacement.

## 4. Technical Selection

**Fig. 2.**
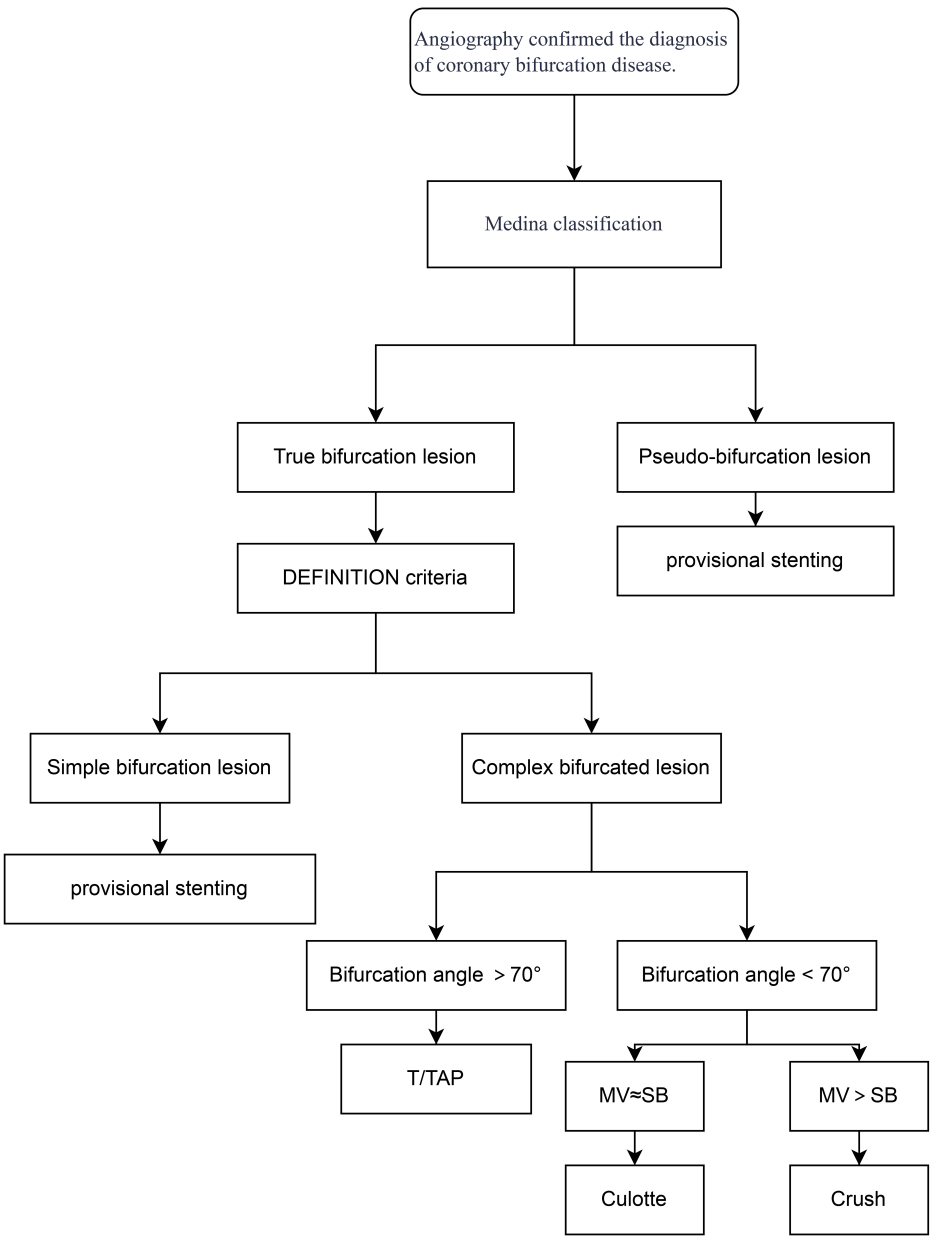
**Flow chart of technical selection**. MV, main vessel; SB, side branch; TAP, T-stent and small protrusion.

When coronary angiography (CAG) identifies CBLs, the lesion is first classified as either a false or true bifurcation according to the Medina classification [10, 11]. For false CBLs, the preferred treatment strategy is PCI with provisional stenting (PS). In cases of true CBLs, risk stratification is performed using the DEFINITION criteria [15]. For simple CBLs, PS remains the recommended approach [24]. However, for complex CBLs, dual-stent techniques are preferred [20]. The selection of the specific dual-stent technique depends on the anatomical characteristics of the bifurcation: (1) T stenting or T-stent and small protrusion (TAP) is recommended when the angle between the MV and SB exceeds 70° [27]. (2) The Culotte stenting is preferred when the angle is <70° and the MV diameter is similar to that of the SB. (3) The Crush stenting is selected when the MV diameter is significantly larger than the SB diameter. A summarized decision-making flowchart for procedural selection is provided in Fig. 2.​ Meanwhile, the calcification of CBLs significantly increases procedural complexity and complication risks in PCI. The presence of calcification not only compromises stent/balloon deliverability but also frequently leads to inadequate stent expansion, consequently elevating the risks of restenosis and stent thrombosis. The assessment needs to combine coronary computed tomography angiography and endovascular imaging to accurately determine both the spatial distribution and severity of calcification. For severely calcified lesions, rotational atherectomy or shockwave balloon angioplasty is recommended for lesion preparation, followed by implantation of high radial strength stents. If necessary, the double-stent technique can be selected. The ultimate treatment strategy should be based on the patient’s hemodynamic status, comorbidities (such as cardiac dysfunction), operator experience, and real-time intravascular imaging guidance to achieve individualized treatment. The comparison of the advantages and disadvantages of different stent technologies is presented in Table 3.

**Table 3.** **Comparative analysis of techniques for CBLs**.

Technique	Advantages	Disadvantages
Provisional stent	1. The operation is relatively easy.	1. Plaque shift or carina shift.
	2. The operation time is relatively short.	2. Risk of branch ostium occlusion and rewiring challenges.
	3. It applies to the majority of lesions.	
T stent	1. Simplified procedure.	1. Potential incomplete branch ostium coverage.
	2. Flexible branch stent positioning.	2. Excessive protrusion increases carina length.
	3. Minimal metal overlap.	3. Requires precise protrusion length control.
Crush	1. Complete coverage of branch ostium.	1. Recrossing challenge and low final KBI success rate.
	2. Immediate dual-branch opening reduces ischemia time.	2. Triple stent layer overlap in the proximal main vessel increases malapposition risk.
		3. It may lead to significant metal carina formation and stent malposition.
		4. Technically complex and time-consuming.
Culotte	1. Optimal branch ostium coverage.	1. Technically complex and time-consuming.
	2. Lowest stent displacement rate.	2. Double stent layer overlap delays endothelialization.
		3. Plaque shift or carina shift may result in guidewire crossing difficulty.
DK-crush/culotte	1. Significantly improved final KBI success rate (nearly 100%).	1. Complex procedural steps.
	2. Reduces metal overlap and improves stent strut apposition.	2. Higher radiation exposure and contrast volume.
	3. Lower restenosis and thrombosis risks.	3. It still depends on the operator’s experience.

Note: DK, double-kissing; KBI, kissing balloon inflation; CBL, coronary bifurcation lesion.

## 5. Challenges and Optimization

### 5.1 Provisional Stent

The provisional stenting procedure consists of the following steps: (1) MV stent implantation; (2) POT of the MV stent; (3) evaluation of SB compromise, with subsequent treatment if required. If branch intervention is needed, the following steps are performed: (4) rewire; (5) SB balloon dilation; (6) reassessment of SB compromise, with stent placement if necessary [28].

**Fig. 3.**
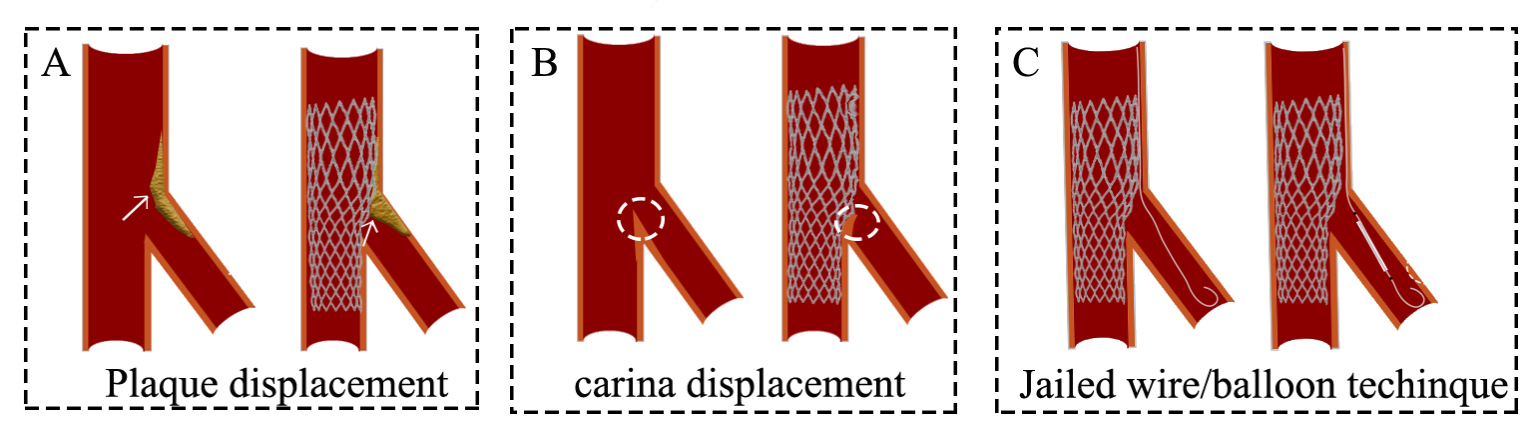
**Challenges and optimization of the provisional stenting technique**. (A) Plaque shift. (B) Carina displacement. (C) Jailed wire or jailed balloon technique. The white arrows represent plaque. The white circles represent the carina.

Challenges and optimization: (1) Plaque displacement (Fig. 3A). It predominantly occurs in lesions with high-volume plaque burden in the pMV; (2) Carina displacement (Fig. 3B). Through intravascular ultrasound (IVUS), it has been found that carinae exhibiting a spiked morphology (“eyebrow sign”) serve as reliable predictors of SB ostial injury following MV stent deployment; Preventive approaches involve protective measures for high-risk SB (V-RESOLVE score ≥12, Table 2), such as JWT or JBT (Fig. 3C), to minimize occlusion risk. Current practice generally recommends branch protection for vessels ≥2.0 mm in diameter. Angiographic assessment frequently proves inadequate for accurate evaluation of SB ostial conditions due to the elliptical geometry of the ostium and potential imaging artifacts from left main plaque, which requires IVUS confirmation. The operator should remember that only 20% of non-left main side branches supply more than 10% of the overall myocardial mass. Therefore, the operator needs to adopt appropriate strategies in combination with the patient’s clinical symptoms and actual situation.

**Fig. 4.**
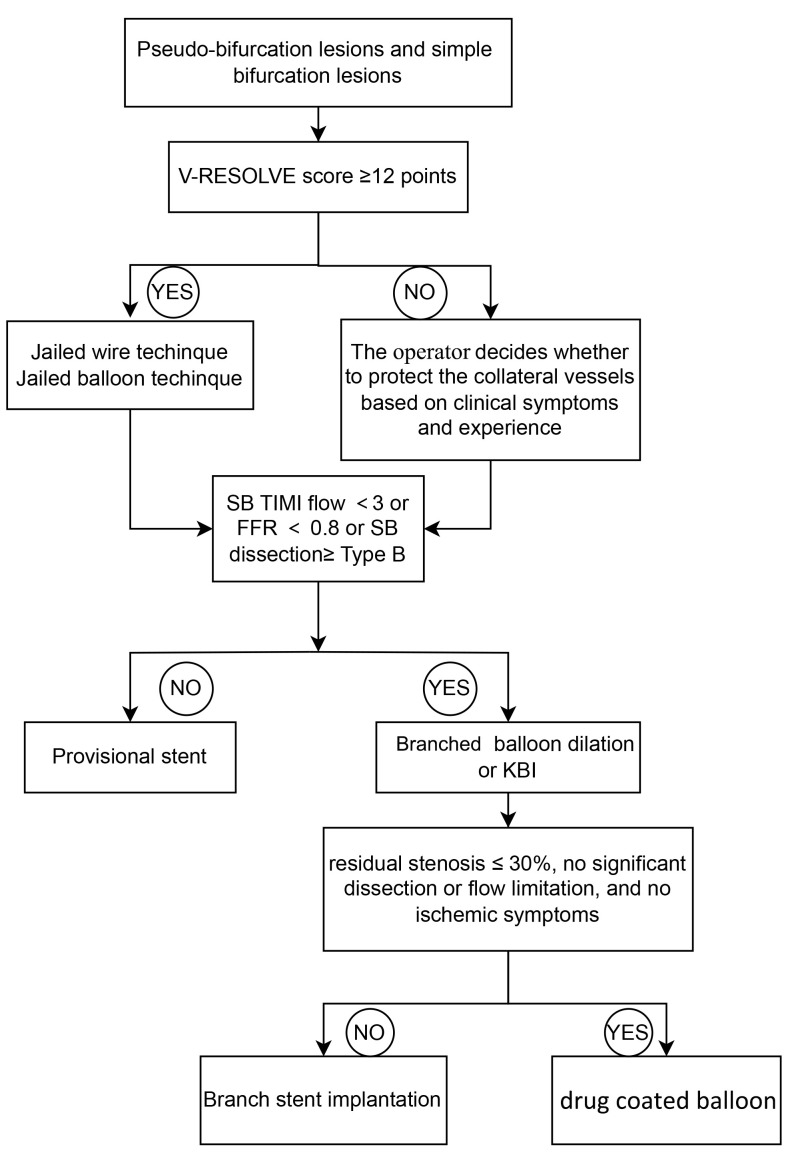
**Flow chart of provisional stenting technique**. SB, side branch; KBI, kissing balloon inflation; TIMI, thrombolysis in myocardial infarction; FFR, fractional flow reserve.

Following MV stenting, corrective measures are taken if SB outcomes are suboptimal (thrombolysis in myocardial infarction (TIMI) flow <3, ≥type B dissection, or fractional flow reserve (FFR) <0.8) [29]. After SB dilation or KBI, a DCB may be used if results are acceptable (residual stenosis ≤30%, no significant dissection or flow limitation, and no ischemic symptoms). Otherwise, SB stenting becomes necessary, converting the procedure to a two-stent technique. The specific technique selection depends on anatomical factors: PS-T/TAP for angles >70° between MV and SB; PS-Culotte for angles <70° with similar vessel sizes; and PS-Crush when the MV diameter exceeds the SB diameter [3]. The flowchart is shown in Fig. 4.

### 5.2 T-stent

**Fig. 5.**
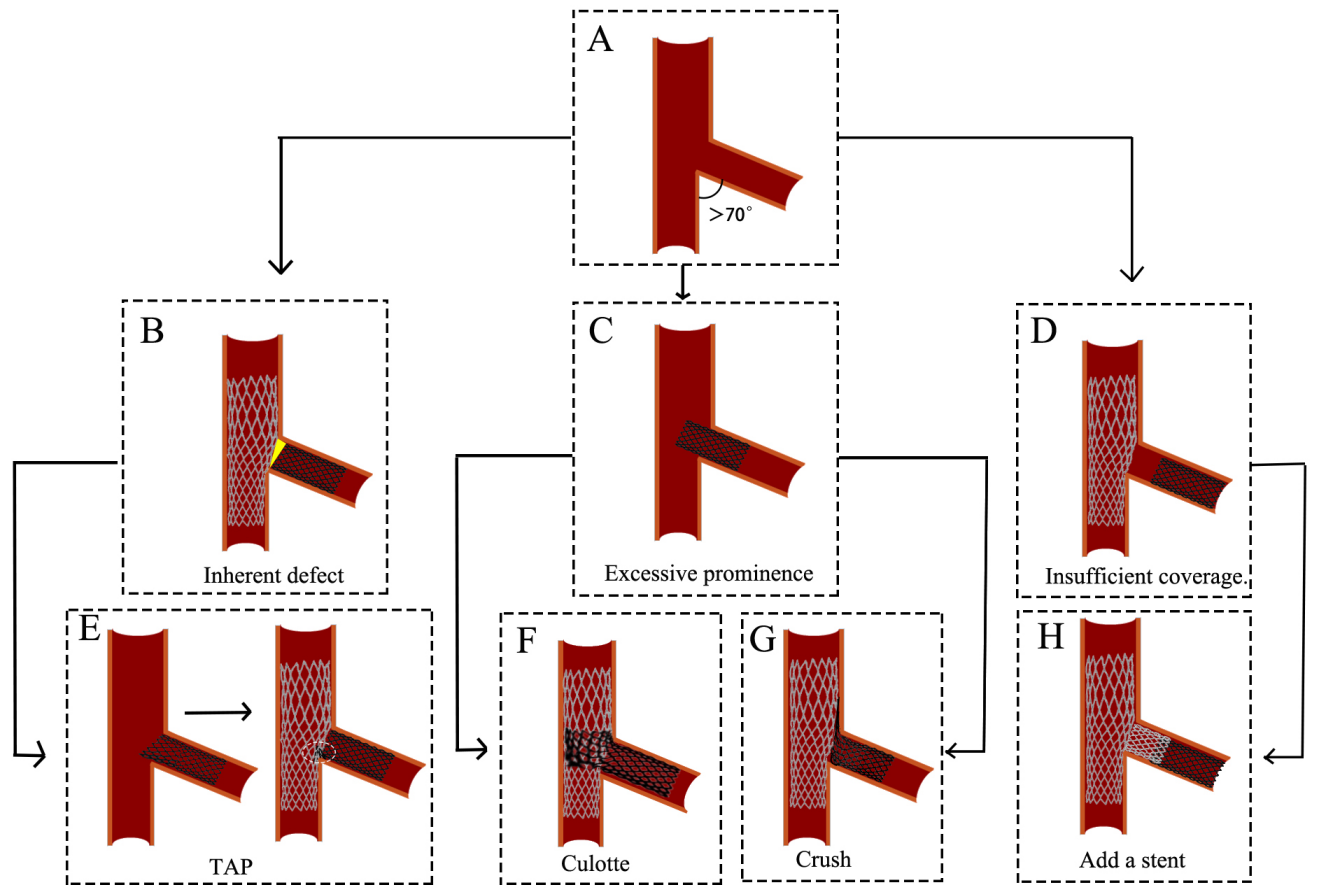
**Challenges and optimization of the T stenting technique**. (A) The angle between MV and SB is greater than 70°. (B) The inherent defect of the T stenting technique. (C) SB stent deployment >2 mm into the MV. (D) Insufficient ostial coverage of SB. (E) A brief process of the TAP technique. (F) The outcome of the Culotte technique. (G) The outcome of the Crush technique. (H) A stent is implanted in the proximal segment of the SB. The yellow square in (B) indicates incomplete coverage of the proximal segment of the SB. The white circles in (E) represent the metallic carina. MV, main vessel; SB, side branch; TAP, T-stent and small protrusion.

When the Angle between MV and SB is greater than 70°, the T-stent technique is selected (Fig. 5A). The T-stenting procedure involves sequential stent placement, beginning with the implantation of a stent in the SB, ensuring coverage of the SB lesion up to its ostium. Subsequently, a stent is deployed in the MV, followed by KBI to optimize stent apposition.

Challenges and optimization: However, a limitation of conventional T-stenting is the frequent incomplete coverage of the SB ostium (Fig. 5B), which contributes to a higher risk of restenosis [27]. To address this issue, the TAP is developed as an optimized approach. In TAP, the proximal edge of the SB stent is intentionally positioned 1–2 mm into the MV, forming a “T” configuration upon deployment [30] (Fig. 5E). This modification ensures complete coverage of the SB ostium. However, TAP introduces a new carina (Fig. 5E), which may influence hemodynamics. Both T-stenting and TAP require precise stent positioning in the SB. Misplacement, whether proximal or distal, can necessitate alternative strategies: (1) If the SB stent is placed proximally (Fig. 5C) and the MV diameter is similar to the SB (MV ≈ SB), conversion to the Culotte technique may be appropriate (Fig. 5F). (2) If the MV is bigger than the SB, the Crush technique can be employed instead (Fig. 5G). (3) If the SB stent is deployed too distally (Fig. 5D), an additional stent may be required to ensure full ostial coverage (Fig. 5H). In order to overcome the positioning problem of traditional technology, Szabo stent technology theoretically achieves precise implantation through an optimized stent anchoring mechanism [31].

### 5.3 Crush

**Fig. 6.**
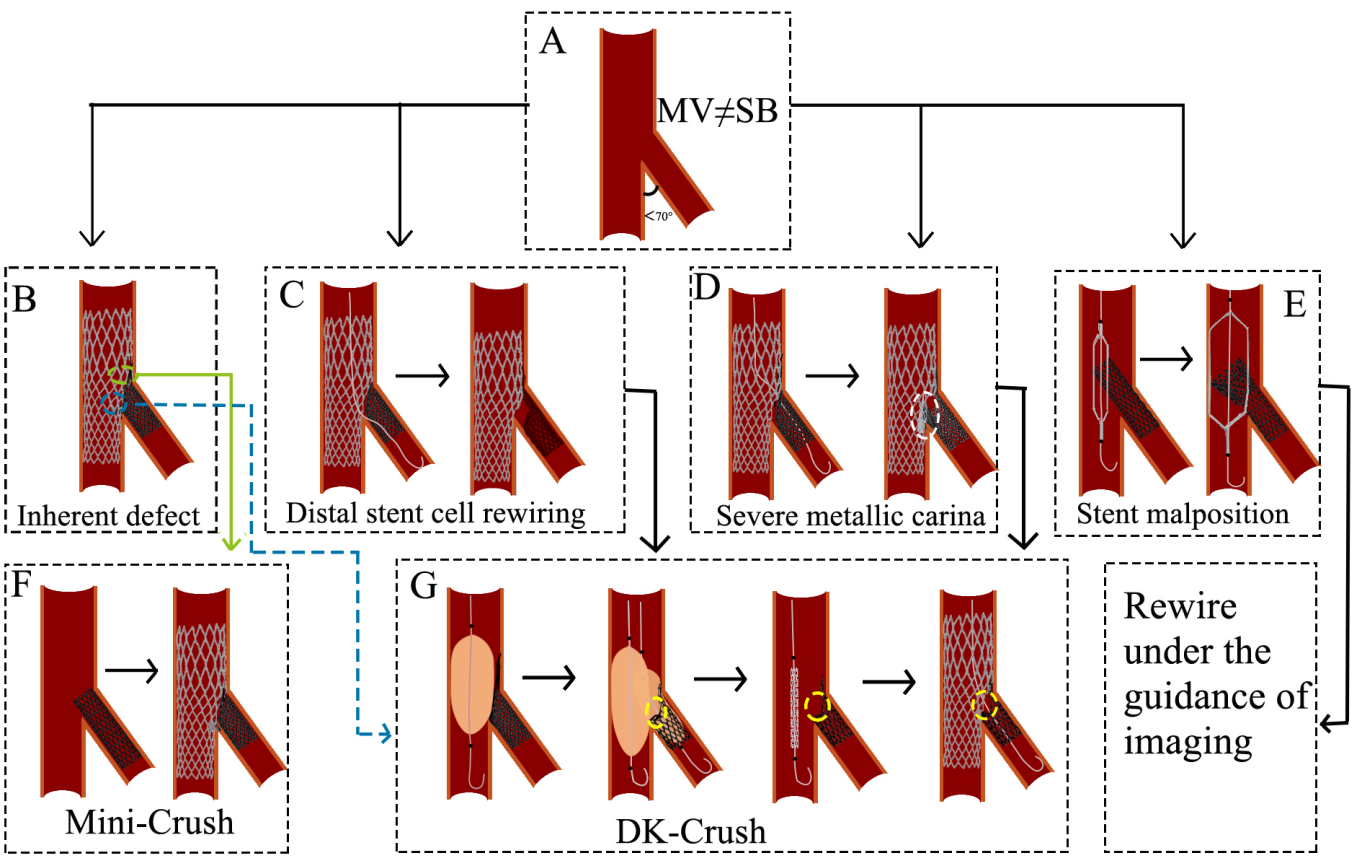
**Challenges and optimization of the Crush technique**. (A) The angle between MV and SB is less than 70°, and they are not equal. (B) The inherent defect of the Crush technique. (C) The wire is passed through the distal stent cell. The subsequent KBI resulted in stent deformation and incomplete lesion coverage. (D) The wire is passed through the proximal stent cell. The subsequent KBI results in the formation of a severe metal carina. (E) The direction of the stent is not controlled during balloon expansion. (F) A brief process of the Mini-Crush technique. (G) A brief process of the DK-Crush technique. The white circle indicates severe metallic carina. The yellow circles represent a single metallic layer. MV, main vessel; SB, side branch; TAP, T-stent and small protrusion; KBI, kissing balloon inflation; DK, double-kissing.

When the angle between MV and SB is less than 70° and they are not equal, the Crush technique is selected (Fig. 6A). The Crush stenting technique involves the simultaneous placement of guidewires in both the MV and SB, followed by stent positioning along each guidewire. The proximal end of the MV stent is positioned to overlap the proximal end of the SB stent. The SB stent is deployed first (approximately 2 mm protrudes into MV), followed by MV stent deployment, which crushes the SB stent against the vessel wall. The guidewire is then advanced through the stent cell into the SB, and balloon dilation is performed to optimize stent expansion, ending up with a final KBI [24].

Challenges and optimization: (1) Stent strut overlap and apposition: The triple-layer stent strut overlap in the MV (Fig. 6B) may result in incomplete stent apposition, increasing thrombosis risk. The Mini-Crush technique minimizes this issue by limiting SB stent protrusion into the MV to 1–2 mm, ensuring complete ostial coverage while reducing metal overlap [32, 33] (Fig. 6F). (2) Rewiring difficulty and low KBI success rate: Rewiring through two stent layers (Fig. 6B) increases procedural complexity and may reduce KBI success, elevating risks of stent thrombosis and in-stent restenosis (ISR). The DK-Crush technique addresses this by performing the first KBI before MV stent implantation, leaving only a single stent layer at the SB ostium and facilitating rewiring [34] (Fig. 6G). The DKCRUSH-I trial demonstrated that final KBI was successfully achieved in 100% of cases using the DK-crush technique, compared to only a 75% success rate in conventional crush technique cases [35]. (3) Suboptimal guidewire passage and stent deformation: Guidewire passage outside the intended stent struts (between the SB stent and vessel wall) can distort stent architecture and compromise lesion coverage [36] (Fig. 6C). The DK-Crush approach emphasizes wire recrossing through the proximal stent struts during initial KBI, minimizing gaps at the ostium [37] (Fig. 6G). (4) Strut malapposition and delayed endothelialization: If the guidewire enters the SB through the proximal stent struts after MV stent deployment, subsequent KBI may elongate metal struts, delay endothelialization, and induce SB stent malapposition (Fig. 6D). The DK-Crush technique resolves this by recrossing the non-distal struts during the second KBI, promoting symmetrical stent expansion and improving strut coverage [34] (Fig. 6G). (5) Unpredictable stent compression direction: The direction of SB stent compression during crushing is often difficult to control (Fig. 6E). Intravascular imaging is recommended to guide precise rewiring and optimize stent positioning.

### 5.4 Culotte

**Fig. 7.**
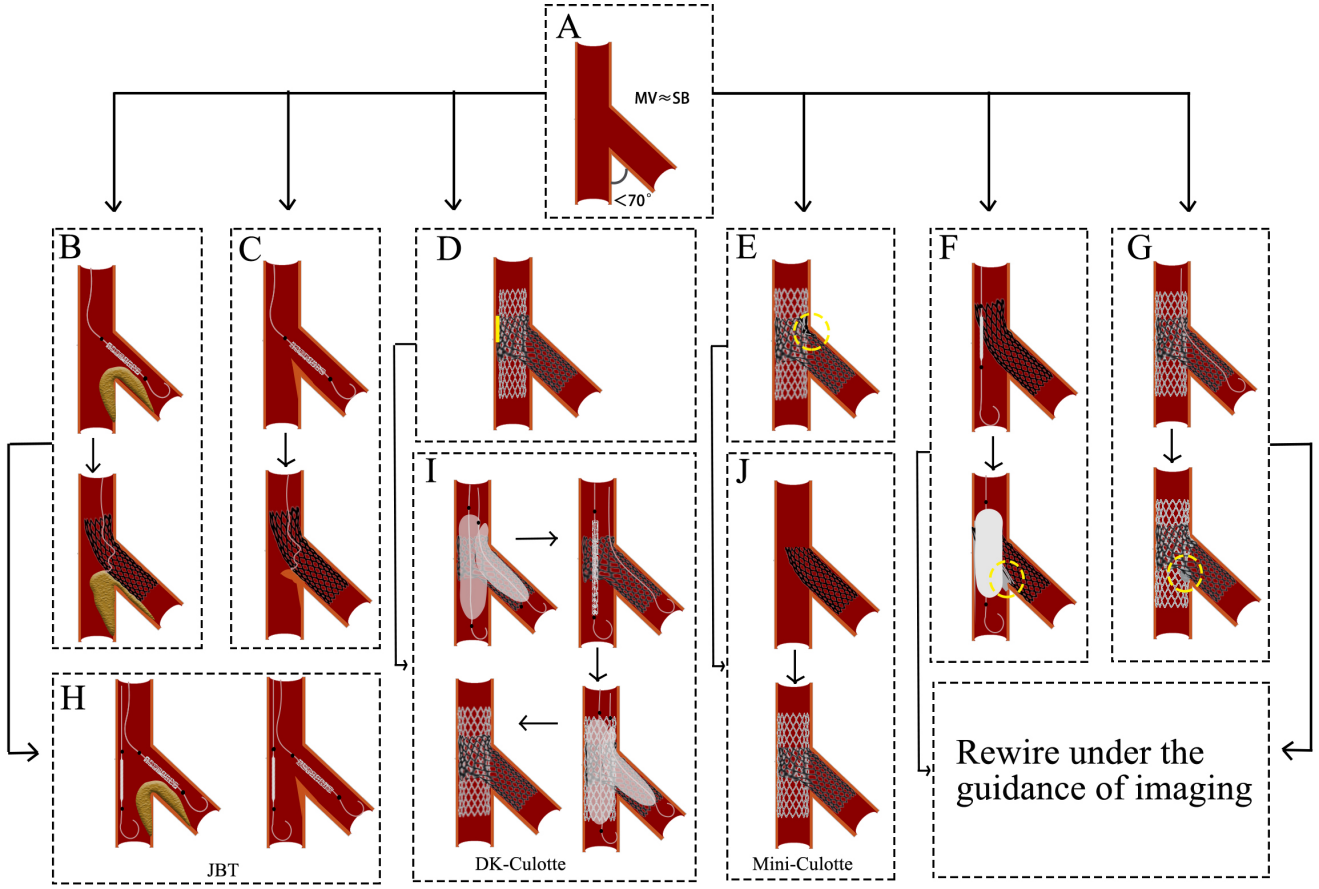
**Challenges and optimization of Culotte technique**. (A) The angle between MV and SB is less than 70°, and they are nearly equal. (B) The plaque shift impedes distal cell rewiring. (C) The carina displacement impedes distal cell rewiring. (D) The MV stent expansion is constrained by the SB stent rings. (E) The MV contains dual layers of stents. (F) The wire is passed through the proximal stent cell. Subsequent balloon expansion results in the formation of a severe metallic carina. (G) The wire is passed through the proximal stent cell. The subsequent KBI resulted in the formation of a severe metallic carina. (H) A protective balloon is implanted in the MV before the SB stent is expanded. (I) A brief process of the DK-Culotte technique. (J) A brief process of the Mine-Culotte technique. The yellow square in (D) indicates poor adherence of the stent. The yellow circle in (E–G) indicates severe metallic carina. MV, main vessel; SB, side branch; JBT, the jailed balloon technique; KBI, kissing balloon inflation; DK, double-kissing.

When the angle between MV and SB is less than 70° and they are nearly equal, the Culotte technique is selected (Fig. 7A). The Culotte stenting technique begins with the implantation of a stent in the SB, with its proximal end extending into the MV. The guidewire is then passed through a distal cell of the SB stent into the distal MV, followed by balloon dilation to expand the stent cell. In the MV, the second stent is subsequently deployed to fully cover both proximal and distal lesions. Finally, the guidewire is recrossed through a distal cell of the MV stent into the SB, where high-pressure balloon dilation and KBI are performed to optimize stent apposition [24].

Challenges and optimization: (1) The guidewire needs to cross through the stent cells twice. Crossing through the proximal cell and the subsequent balloon expansion may lead to the formation of a metallic carina (Fig. 7F,G), requiring the operator to possess proficient wire-manipulation skills. (2) After SB stent deployment, plaque shift (Fig. 7B) or carina displacement (Fig. 7C) may exacerbate MV stenosis and impede distal cell rewiring. Pre-embedding a protective balloon in the MV (Fig. 7H) allows immediate lumen restoration upon rewiring failure. (3) The SB stent rings may constrain MV stent expansion, leading to poor adhesion of the stent to the vascular wall (Fig. 7D). The degree of MV stent expansion is determined by the SB stent rings. The DK-Culotte modification addresses this through dual KBI (Fig. 7I), ensuring optimal stent expansion and alignment [38, 39]. A bench study demonstrated that the DK Culotte technique optimizes stent apposition through sequential KBI [40]. (4) Dual-layer stent overlap in the MV increases the risk of restenosis (Fig. 7E). The mini-Culotte technique (Fig. 7J) minimizes stent overlap, reducing vessel irritation and restenosis potential [41].

## 6. Application of DCB in CBLs

DCB is an angioplasty device coated with antiproliferative agents (e.g., paclitaxel, sirolimus) that transfers the drug to the vessel wall during balloon inflation, inhibiting vascular smooth muscle cell proliferation and migration to reduce restenosis. DCB has emerged as an attractive alternative strategy for treating coronary ISR, small vessel disease, and CBLs [42]. Particularly in small vessel lesions with diameters ≤2.75 mm, DCB demonstrates clinical outcomes non-inferior to stents [43]. This approach simplifies the procedure and reduces stent-related complications while offering the advantage of leaving no permanent implant [44], consequently shortening the required duration of dual antiplatelet therapy. However, current research remains limited regarding DCB application in branches exceeding 2.75 mm in diameter, with insufficient evidence supporting the safety of DCB in larger diameter lesions, necessitating further investigation. When treating CBLs with DCB, two predominant strategies exist: deployment of drug-eluting stents in the MV combined with DCB in the SB, or exclusive DCB utilization in both MV and SB. For non-true CBLs, standalone DCB therapy is generally employed, whereas true CBLs typically warrant a main-branch drug-eluting stent with side-branch DCB as the conventional approach. Recent studies demonstrate that DCB treatment following coronary atherectomy for CBLs—including those involving the left main coronary—can reduce stent requirements, obviate complex stenting techniques, and yield favorable clinical outcomes. Although DCBs offer significant advantages, their use may be suboptimal or even contraindicated in specific clinical scenarios (Table 4) [45, 46].

**Table 4.** **Contraindications for DCB strategy**.

Category	Specific considerations.
Severely calcified lesions	May impair balloon expansion or drug effect (requires adjunctive plaque modification techniques).
High-risk vessels	Ostial angle >70° with diameter ≥2.5 mm (high elastic recoil risk);
	Severe vessel tortuosity/angulation;
	Heavy thrombus burden.
Flow-limiting	Persistent slow-flow or TIMI flow <grade 3.
Others	Hypersensitivity to coating drugs (e.g., paclitaxel/sirolimus).

Note: TIMI, thrombolysis in myocardial infarction; DCB, drug-coated balloon.

Challenges and optimization: (1) Before DCB angioplasty, the desired outcomes of lesion preparation include residual stenosis ≤30%, absence of significant dissection or flow-limiting complications, and no ischemia-related symptoms. If suboptimal results are observed, emergency stent implantation may be performed to prevent procedural complications [23]. (2) Insufficient inflation duration (<30 seconds) or excessive pressure (>nominal pressure) may elevate dissection risk; (3) Non-uniform distribution of anti-proliferative agents within the vessel wall. Coping strategy: Prolonged low-pressure inflation (60–90 seconds at nominal pressure) to ensure adequate drug transfer; Strict 1:1 device-to-artery diameter ratio for DCB selection. The successful implementation of DCB angioplasty relies on meticulous lesion evaluation and appropriate procedural techniques.

## 7. Application of Imaging and Functional Assessment in CBLs

The conventional assessment of CBLs predominantly relies on CAG. However, PCI for CBLs typically requires more complex stent implantation techniques and is associated with higher risks of mortality, myocardial infarction, and repeat revascularization compared to non-CBLs. Sole reliance on CAG has inherent limitations, including suboptimal evaluation of lesion characteristics and stent implantation outcomes [47].

The 2024 European Society of Cardiology (ESC) Guidelines for the Management of Chronic Coronary Syndromes have assigned a Class IA recommendation for IVUS and optical coherence tomography (OCT) guidance in PCI for true CBLs [48]. These imaging modalities play a pivotal role in optimizing stent sizing, assessing plaque distribution, guiding wire recrossing, and confirming stent apposition. Thereby, it significantly reduces the risk of post-procedural cardiovascular adverse events. The OCTOBER trial demonstrated that OCT-guided PCI significantly reduced 2-year MACE compared with angiography-alone guidance (10.1% vs. 14.1%, HR = 0.70, *p* = 0.035), with particular advantages in minimizing branch occlusion and stent thrombosis. Similarly, the ULTIMATE trial’s 3-year follow-up showed significantly lower target vessel failure in the bifurcation subgroup with IVUS guidance (HR 0.48, 95% CI: 0.27–0.87) [49]. The 5-year outcomes from DKCRUSH-II revealed reduced myocardial infarction rates in patients undergoing IVUS assessment (1.8% vs. 5.4%; *p* = 0.043) [50].

From a technical perspective, IVUS provides quantitative assessment of plaque burden (>50% indicating high risk for branch occlusion) and calcification arc; While OCT’s superior resolution enables precise identification of thin-cap fibroatheroma, lipid core extent, and calcification thickness. OCT additionally allows prediction of branch occlusion risk through measurements of bifurcation carina angle or distance from the proximal branch point to the carina tip. The choice between these modalities requires careful consideration of cost, availability, and patient-specific factors [51]. IVUS maintains advantages in wider availability, lower cost (approximately 1/2 to 1/3 of OCT consumable expenses), and no need for flow occlusion, making it particularly suitable for primary hospitals and economically constrained patients [52]. Conversely, OCT’s ultra-high resolution makes it ideal for complex lesions such as left main bifurcations, where it excels in detecting stent edge dissections and tissue prolapse with unparalleled precision. The meta-analysis found that OCT-guided and IVUS-guided stent implantation outperformed angiography, with OCT excelling in stent apposition assessment [51]. However, its clinical adoption is limited by higher equipment costs. While OCT represents the optimal choice for precision-oriented centers, IVUS remains the more cost-effective alternative in resource-limited settings, with both modalities demonstrating complementary roles in contemporary bifurcation PCI practice. The decision should ultimately be individualized based on lesion complexity, institutional capabilities, and economic considerations, with both techniques offering substantial improvements over angiography-alone guidance as evidenced by multiple randomized trials and meta-analyses.

Functional assessment of CBLs has emerged as a pivotal tool for precision interventional decision-making by quantifying hemodynamic impairments, overcoming the anatomical limitations of conventional angiography [53]. Its fundamental value lies in accurate ischemia risk stratification: FFR measurements reveal that 72% of SB with ≥75% angiographic stenosis demonstrate FFR >0.80, indicating no functional ischemic significance. Avoiding unnecessary SB interventions in such cases significantly reduces the risks of restenosis and stent thrombosis [54]. During MV stent implantation, direct FFR measurement of the SB using the “jailed pressure wire technique” may preclude the need for provisional stenting when values exceed 0.80 with TIMI grade 3 flow, thereby simplifying the procedure and reducing complications [55]. Beyond FFR, instantaneous wave-free ratio (iFR) achieves adenosine-free assessment through diastolic pressure gradient analysis (cutoff ≤0.89), demonstrating 94% diagnostic concordance with FFR [56]. Resting full-cycle ratio (RFR) and quantitative flow ratio (QFR) derived from coronary angiography provide additional non-invasive alternatives [57]. The integration of OCT/IVUS with functional data (“anatomo-functional fusion technology”) enables precise localization of high-risk plaques exhibiting thin-cap fibroatheroma with functional ischemia, allowing targeted intervention [58].

## 8. Other Techniques for Interventional Treatment of CBLs

Recent innovations in interventional cardiology are revolutionizing treatment strategies for CBLs. Bioresorbable scaffolds have emerged as a promising option, offering complete biodegradation, restoration of vasomotion, and reduced long-term inflammatory responses from permanent metallic implants [59]. Clinical studies have demonstrated favorable outcomes when applying Bioresorbable scaffolds to CBLs. The BioMime™ Branch sirolimus-eluting coronary side-branch stent features a unique design with four radiopaque markers [8]: proximal/distal markers, side-branch ostium marker, and carina marker. It has garnered increasing attention. Additionally, the R-One robotic system for PCI is gaining traction [9]. While requiring further clinical validation, these technologies represent significant advances toward personalized, physiology-guided treatment approaches. With accumulating evidence, these innovations may establish new paradigms for precision therapy of CBLs.

## 9. Discussion 

CBLs remain a challenging area in PCI. This complexity stems from both anatomical heterogeneity, intricate procedural considerations and long-term clinical outcomes. This review systematically examines current key techniques, strategy optimization approaches, and individualized management paradigms for CBLs, incorporating advances in functional assessment and intravascular imaging. Initially, procedural strategy selection constitutes the core challenge. The single-stent strategy (e.g., provisional stenting) is preferred for pseudo-bifurcation lesions due to procedural simplicity. However, its risk of SB occlusion cannot be overlooked, particularly in high-risk patients (V-RESOLVE score ≥12) [60]. Dual-stent techniques (e.g., Crush, Culotte, T/TAP) improve SB coverage but warrant vigilance regarding metallic carina formation, stent malapposition, and late thrombosis risks. Modified techniques, including DK-Crush and DK-Culotte enhance procedural success and long-term outcomes through optimized wire recrossing and final KBI. But their technical complexity demands greater operator expertise [60, 61]. Besides, functional assessment (e.g., FFR/RFR) and intravascular imaging (IVUS/OCT) provide critical guidance for precision intervention; The functional evaluation identifies hemodynamically insignificant SB lesions to avoid unnecessary intervention. And intravascular imaging optimizes stent sizing, plaque characterization, and apposition assessment, significantly reducing adverse events—a benefit demonstrated in trials such as OCTOBER and ULTIMATE [62]. Finally, the emerging technologies, including DCB, bioresorbable scaffolds, and [63], offer novel therapeutic possibilities. DCB minimizes stent implantation while shortening dual antiplatelet therapy duration, which is particularly advantageous for small-vessel disease [64]. The bioresorbable scaffolds address long-term metallic stent retention concerns [65]. However, the long-term safety and efficacy of these techniques still require further validation through robust clinical evidence. Consequently, individualized strategies integrating anatomical characterization, functional assessment, and intravascular imaging guidance are essential for bifurcation intervention. Future research should prioritize refinement of stent techniques, functional-imaging hybrid approaches, and clinical validation of emerging technologies to enable safer, more precise therapeutic algorithms for patients with bifurcation lesions.

## 10. Conclusion

The treatment of CBLs remains challenging due to issues like plaque shift, stent strut obstruction, and incomplete stent apposition. Current strategies rely on Medina classification, DEFINITION criteria, and V-RESOLVE scoring to guide individualized approaches. Key techniques include precise lesion localization, accurate rewiring, and dual kissing balloon inflation under intravascular imaging guidance, which enhance procedural success and long-term outcomes while minimizing complications. However, real-world application requires clinical flexibility, as optimal management continues to evolve and demands operator expertise in adapting to specific lesion characteristics.

## Abbreviations

CBL, coronary bifurcation lesion; PCI, percutaneous coronary interventions; MACE, major adverse cardiovascular events; pMV, proximal main vessel; dMV, distal main vessel; MV, main vessel; SB, side branch; V-RESOLVE, Visual Risk Prediction of Side-branch Occlusion in Coronary Bifurcation Intervention; DK-crush, double-kissing crush; JWT, jailed wire technique; JBT, jailed balloon technique; POT, proximal optimization technique; KBI, kissing balloon inflation; PS, Provisional stenting; TAP, T-stent and small protrusion; ISR, in-stent restenosis; DCB, drug-coated balloon; ISR, in-stent restenosis; CAG, coronary angiography; IVUS, intravascular ultrasound; OCT, optical coherence tomography; TIMI, thrombolysis in myocardial infarction; FFR, fractional flow reserve; iFR, instantaneous wave-free ratio; RFR, resting full-cycle ratio; QFR, quantitative flow ratio.
